# Cognitive-Behavioral Therapy−Based Massed Brief Psychoeducational Group via Videoconference for Social Anxiety: Randomized Controlled Trial

**DOI:** 10.2196/79825

**Published:** 2026-01-06

**Authors:** Lele Feng, Wei Liu, Liechuan Cui, Deborah Dobson, Xinfeng Tang

**Affiliations:** 1 Department of Psychology, Renmin University of China, Huixian Building, 9th, 59 Zhongguancun St., Haidian District, Beijing, 100082, China, 86 13581993126; 2 Department of Psychology, University of Calgary, Calgary, AB, Canada

**Keywords:** cognitive behavioral therapy, massed intervention, psychoeducational group, randomized controlled trial, social anxiety, workshop intervention, low-intensity intervention

## Abstract

**Background:**

Group cognitive-behavioral therapy (CBT), delivered through weekly videoconference sessions, has been shown to effectively reduce social anxiety. However, no studies have evaluated CBT delivered via videoconference in a 2-day massed brief psychoeducational group format.

**Objective:**

This randomized controlled trial aimed to evaluate the efficacy of a videoconferencing CBT−based massed brief psychoeducational group among Chinese university students with social anxiety.

**Methods:**

University students with social anxiety were recruited online and randomly assigned to an intervention group or a waitlist control group. Participants in the intervention group attended a 2-day workshop via videoconference. Assessments were conducted at baseline (T1), posttest (T2), 1-month follow-up (T3), and 3-month follow-up (T4), using the Social Phobia Inventory, Brief Fear of Negative Evaluation Scale, Depression Anxiety Stress Scales–Short Form, Social Anxiety Knowledge Test, Social Anxiety Stigma Inventory, and Attitudes Toward Seeking Professional Psychological Help Scale–Short Form.

**Results:**

The intervention group showed significant reductions in Social Phobia Inventory scores (*β*=−4.00, 95% bootstrap CI −6.55 to −1.22; *d*_T2-4_=−0.97 to −0.81) and Brief Fear of Negative Evaluation Scale scores (*β*=−1.37, 95% bootstrap CI −2.64 to −0.08; *d*_T3_=−0.56), as well as significant increases in Social Anxiety Knowledge Test scores (*β*=.62, 95% bootstrap CI 0.05-1.17; *d*_T2-4_=0.86-1.53). No significant changes were observed in Depression Anxiety Stress Scales–Short Form, Social Anxiety Stigma Inventory, or Attitudes Toward Seeking Professional Psychological Help Scale–Short Form scores.

**Conclusions:**

The findings indicate that videoconferencing CBT−based massed brief psychoeducational group was effective in reducing social anxiety among university students. Future research with larger and more diverse samples is recommended to validate the efficacy and assess the scalability of this intervention format.

## Introduction

Social anxiety refers to the intense fear, worry, or avoidance of social interactions and situations in which one may be evaluated by others [1]. When social anxiety reaches a severity that impairs daily functioning, it is diagnosed as social anxiety disorder or social phobia [2]. A meta-analysis reported that the prevalence of social anxiety symptoms among Chinese adolescents and young adults aged 15‐25 years is 29.8% [3]. Social anxiety disorder can lead to functional impairments across multiple aspects, including work, academics, social functioning, and cognitive processes [4]. Even subclinical symptoms, which do not meet diagnostic criteria, can negatively impact various aspects of life [5] and may progress into a chronic and debilitating condition if left unaddressed [6]. For example, social anxiety may hinder college students’ learning, decrease their well-being [7], and even elevate their risk of suicidal thoughts or behaviors [8]. Early intervention is, therefore, critical to alleviating symptom severity and the likelihood of developing social anxiety disorder.

Cognitive-behavioral therapy (CBT) is widely recognized as the gold standard for treating social anxiety [9]. Meta-analytic findings indicate that compared to pharmacological and alternative therapeutic approaches, CBT demonstrates superior efficacy, greater safety, and lower relapse rates in both the short and long term, making it the most highly recommended treatment [10,11]. CBT is typically delivered in individual or group formats, both of which have shown positive effects on social anxiety. However, group CBT has its unique advantages by allowing participants to engage with unfamiliar peers in socially relevant contexts while benefiting from mutual support. It also enables more individuals to receive treatment within a given timeframe [12]. Therefore, group interventions are also frequently used in both research and clinical practice.

In terms of therapist-guided delivery medium, psychological interventions can be categorized into face-to-face CBT and remote CBT. Traditional group CBT typically involves therapists working face-to-face with participants; however, limited mental health resources often prevent individuals from accessing CBT or other mental health services in their daily lives [13]. Moreover, traditional group CBT may entail several temporal and locational constraints, limiting the delivery of timely clinical interventions [14]. In this context, internet-based remote CBT offers a viable alternative [15]. Among remote modalities, *videoconferencing* is commonly used, as it enables synchronous, real-time communication through audio and video and allows therapists and participants to interact directly from different locations. Compared to other digital formats, such as message-based or app-based interventions, videoconferencing CBT maintains therapist visibility, facilitating greater interaction and engagement between participants and therapists [16]. Studies have shown that videoconferencing CBT yields significant therapeutic effects for social anxiety and social anxiety disorder, with symptom improvements maintained for up to 3 months postintervention [17,18] and effect sizes ranging from medium to large [19]. Moreover, compared to traditional CBT, videoconferencing CBT may alleviate the resistance to therapy triggered by face-to-face interaction in individuals with social anxiety [20].

Low-intensity interventions have become a common approach in the delivery of psychological interventions. The National Institute for Health and Care Excellence recommends that group CBT for social anxiety be delivered in weekly sessions, for a total of 8‐12 sessions [21]. This implementation of full protocol requires substantial time and financial resources; as such, low-intensity CBT has emerged to meet the growing demand for mental health services while ensuring treatment effectiveness [22]. Within health care systems, low-intensity interventions are commonly employed in primary care settings for adults experiencing symptoms of depression or specific phobias [23]. A meta-analysis revealed that low-intensity CBT produced large effect sizes in the treatment of anxiety disorders (*d*=1.06) [24].

A common form of low-intensity intervention involves employing only select core therapeutic techniques rather than the full treatment protocol; this approach is often referred to as a *brief* intervention. For example, in social anxiety interventions, Clark’s full protocol typically includes components, such as attention training, video feedback, behavioral experiments, and discrimination training [25]. However, Heimberg’s intervention full protocol incorporates multiple sessions of cognitive restructuring and graduated exposure. Brief interventions for social anxiety based on these 2 protocols have also demonstrated promising outcomes [26]. A group CBT for children with social anxiety, delivered in three weekly 3-hour sessions, included psychoeducation, cognitive strategies, and behavioral exposure. Significant reductions in participants’ social anxiety scores were observed at both posttest and 3-week follow-up [27]. A recent 7-day internet−based CBT program for social anxiety disorder also demonstrated substantial outcomes (Hedges *g*s=1.26‐1.9). This program consisted of 6 online lessons accompanied by practice tasks. Participants were required to complete lessons and corresponding exercises, which included exposure tasks, cognitive challenges, and communication skills practice [28]. More recently, a 1-day CBT−based workshop consisting of cognitive restructuring and assertiveness training for secondary vocational students demonstrated moderate effect sizes in reducing social anxiety symptoms [29].

Another common type of low-intensity intervention is the psychoeducational group. According to Gladding’s classification, group interventions are generally divided into *psychoeducational* groups and *counseling* groups [30]. Compared to counseling groups, psychoeducational groups are lower in intensity and emphasize using educational methods to acquire information and develop related meaning and skills [31]. These groups integrate both knowledge acquisition and skill development, efficiently delivering information about psychological disorders while also providing opportunities for practice and experiential learning. Accordingly, 1 major aim of psychoeducational groups is to enhance participants’ mental health literacy, which is referred to as the ability to recognize a disorder, understand it, reduce stigma, and seek appropriate psychological help [32]. In addition, the skills training component can help alleviate psychological distress. Evidence indicates that psychoeducational interventions are effective in alleviating anxiety symptoms, including social anxiety [33,34]. A further advantage of psychoeducational groups is their ability to accommodate a larger number of participants. Counseling groups for social anxiety typically have limited membership, with recommended group sizes ranging from 6 to 12 participants [26,35]. In contrast, psychoeducational groups are often larger in scale, commonly ranging from 15 to 40 participants [30].

In terms of intervention frequency, the conventional model involves *spaced* interventions (eg, 1 session per week). Alternatively, *massed* intervention compresses the treatment timeline by delivering multiple sessions over a relatively short timeframe. Meta-analytic evidence indicates that for anxiety disorders in young adults, massed CBT can achieve intervention outcomes comparable to those of full-protocol CBT [36]. For example, Deacon and Abramowitz [37] conducted a 2-day CBT for panic disorder, condensing a 12-session protocol into 2 sessions of 6 and 3 hours, respectively. The intervention included psychoeducation, therapist-assisted exposure, fear hierarchy construction, and in vivo exposure. Following the treatment, all 10 participants showed significant reductions on the Panic Disorder Severity Scale, with large effect sizes (*d*_s_=0.96‐3.29). To date, spaced brief CBT has been shown to effectively reduce social anxiety and related negative beliefs [38,39]; however, no empirical evidence currently exists on the effects of massed brief CBT for social anxiety.

The vast majority of existing remote interventions for social anxiety employ spaced, full-protocol treatment programs, typically consisting of 8 or 12 weekly sessions. These interventions are generally conducted in groups of 5‐12 participants [40-42]. While this model offers several advantages, it also presents challenges. For example, participants may find it difficult to sustain long-term engagement remotely, which can result in them dropping out. A meta-analysis reported that the dropout rate for group CBT interventions targeting anxiety disorders is 24.6% [43]. To alleviate social anxiety more rapidly, efficiently, and conveniently, we developed a low-intensity intervention format—the massed brief psychoeducational group (MBPG)—delivered via videoconference. This study aimed to evaluate the effects of this delivery model on university students with social anxiety through a randomized controlled trial. We hypothesized that compared to the waitlist control group, participants in the intervention group would show significant reductions in social anxiety, fear of negative evaluation, and depressive symptoms following treatment. Additionally, we expected improvements in social anxiety literacy, including increased knowledge of social anxiety, reduced social anxiety stigma, and more positive attitudes toward seeking professional psychological help.

## Methods

### Participants

According to a meta-analysis by Mayo-Wilson et al [10], interventions with shortened sessions based on the Clark and Wells model for social anxiety have demonstrated large effect sizes. Based on these findings, the expected effect size for this study was conservatively set at 0.80. A priori power analysis using the G*Power 3.1 by Faul et al [44] indicated that a total sample size of 52 would be required. Accounting for an anticipated dropout rate of 20%, the target sample size was set at 65. Accordingly, a minimum of 65 participants were targeted.

Participants were recruited online and were required to meet the following eligibility criteria: (1) current enrollment in a university program, with no restrictions on degree level; (2) availability to attend a 2-day remote workshop and willingness to be randomly assigned; (3) no suicidal ideation and no formal diagnosis of any psychiatric disorder; (4) no psychotropic medication use within the past year and no history of psychological treatment or counseling; and (5) no specific cutoff on social anxiety scores was required for inclusion. As a psychoeducational group, our primary goal was to reach individuals who experienced social anxiety−related difficulties and were motivated to make changes, regardless of their symptom severity. Therefore, although a cutoff value of the Social Phobia Inventory (SPIN) is commonly used to indicate clinically significant symptoms, we did not adopt this cutoff during recruitment. Individuals were eligible for this workshop as long as they perceived themselves affected by social anxiety. Despite the absence of a formal cutoff, participants’ baseline levels of social anxiety were relatively high. Among those enrolled, 62 (90%) of the 69 participants scored above the clinical threshold of 19 on the SPIN, with a mean score of 37.91 (SD=13.67).

Participants were randomly assigned to either the intervention group (n=39) or the waitlist control group (n=39). Prior to the start of the intervention, 7 participants from the intervention group and 2 from the control group withdrew. A total of 69 participants ultimately took part in the study (n=32 in the intervention group; n=37 in the control group).

### Ethical Considerations

This study received approval from the Ethical Review Committee of the Department of Psychology at Renmin University of China (IRB-24‐041). All participants provided written informed consent before participating. Individuals who completed all 4 assessments received both monetary compensation of 40 RMB (US $ 5.70) in total (10 RMB [US $1.42] per assessment) and a commemorative gift. To ensure the protection of participants’ privacy, the raw data were accessible only to the research team and numerical IDs were assigned in lieu of personally identifiable information during data handling. All reported results were fully anonymized, and no identifiable personal features are presented in the manuscript.

### Procedure

Simple randomization was employed to assign participants to groups. Random sequences were generated using Microsoft Excel, with half of the participants allocated to the intervention group and the other half to the waitlist control group.

Before the intervention, an online questionnaire was administered to all participants to collect baseline data (T1). The posttest (T2) was conducted 1 week after the intervention, with follow-up assessments at 1 month (T3) and 3 months (T4) postintervention. Participants in the waitlist control group received the same 2-day online workshop intervention as those in the intervention group after all data collection was completed.

### Intervention

This study implemented a CBT-based MBPG intervention, delivered via videoconferencing. The intervention was delivered synchronously via Tencent Meeting, an online platform similar to Zoom. All needed materials were presented in the form of slides shared on screen during the online sessions. The intervention program was named *Joymaster Workshop* to engage participants’ interest, reduce associated stigma, and symbolize mastery of social enjoyment. For CBT-based counseling groups targeting social anxiety, a recommended group size is approximately 6 participants [45], whereas psychoeducational groups are typically larger [30]. To avoid reductions in interaction, experiential engagement, and opportunities for practice associated with overly large groups, we set the size of the psychoeducational group at 15‐20 participants. Therefore, we divided the 32 participants in the intervention condition into 2 workshops. In allocating participants, we considered their time availability and aimed to maintain balance across the 2 groups in terms of size and gender distribution to minimize potential biases arising from group composition. The 2 workshops ultimately included 17 and 15 participants, respectively. Both workshops were delivered by the same group leaders on 2 consecutive weekends, with identical content to ensure treatment fidelity. The timing of the post-intervention and follow-up assessments was adjusted according to the specific workshop each participant attended.

The term *massed* refers to the delivery format, which consisted of 2 consecutive days, a total duration of 12 hours. As a *brief* intervention, the program selectively incorporated effective techniques from full-protocol CBT, focusing on cognitive restructuring and behavioral experiments. Contemporary CBT interventions for social anxiety typically follow either the Heimberg protocol or the Clark protocol [21]. Given the massed format and the aim of achieving effective outcomes within a short timeframe, this study’s program prioritized cognitive change over habituation through exposure, as the latter generally requires more extended treatment. Therefore, we adapted the cognitive restructuring technique from Hope and Heimberg’s model, which involves challenging automatic thoughts using supporting and opposing evidence [45].

Behavioral experiments were based on the approach by Leigh and Clark [46] as well as Hofmann and Otto [47], in which participants design and engage in social tasks to test and challenge their automatic thoughts. Behavioral experiments also enhanced the interactivity of the massed intervention and promoted participant engagement. A summary of the main intervention modules is presented in Table 1. Among these, the out-of-session behavioral experiments required participants to complete a selected task during a 2-hour lunch break, based on plans developed at the end of the morning session. Examples included asking a passerby to take a photo of them with a trash can or initiating a brief conversation with someone new in the campus canteen. All participants completed a behavioral experiment.

This intervention was conducted as a *psychoeducational* group rather than a counseling group. First, we employed online slide presentation as visual aids, combining didactic instruction for efficient knowledge delivery with opportunities for experiential practice of therapeutic techniques. Second, the intervention accommodated a larger number of participants than is common in counseling groups, with each workshop hosting 15‐20 individuals.

**Table 1.** Intervention modules.

Module	Techniques	Contents
Day 1—Morning	Psychoeducation	Introduce the concept of social anxiety: definition, prevalence, diagnostic criteria, typical age of onset, and negative impacts Provide an overview of cognitive models and contributing factors Introduce treatment approaches for social anxiety
Day 1—Afternoon	Cognitive restructuring (Part 1)	Practice the first 2 steps of cognitive restructuring: Identify automatic thoughts Identify cognitive biases
Day 2—Morning	Behavioral experiments	Explain the purpose and steps of behavioral experiments Conduct in-session behavioral experiments Design out-of-session behavioral experiments for lunch break
Day 2—Afternoon	Cognitive restructuring (Part 2)	Reflect on behavioral experiments conducted during lunch break Practice the final 2 steps of cognitive restructuring Challenge automatic thoughts Develop realistic, rational thoughts Develop an action plan and conclude the session

### Treatment Fidelity

The intervention was delivered by a leader and an assistant. The leader was a PhD-level counselor intensively trained in CBT, and the assistant was a master’s student in psychology. Psychoeducational content was presented primarily through PowerPoint slides, with all procedures structured around the slide content. This ensured a highly standardized delivery and strong adherence to the intervention protocol.

### Measurements

#### Primary Outcome—Social Anxiety

The Chinese version of the 17-item SPIN, developed by Connor et al [48] and revised by Xiao et al [49], was used to assess the severity of social anxiety. The scale comprises 3 subscales: fear (6 items; eg, fear of embarrassment or rejection), avoidance (7 items; eg, avoiding social situations because of fear), and physiological symptoms (4 items; eg, blushing or sweating in social settings)—covering the core clinical manifestations of social anxiety. It uses a 5-point Likert rating scale from 0 (*not at all*) to 4 (*extremely*), with higher scores indicating greater levels of social anxiety. In this study, the SPIN had a Cronbach *α* of 0.94 at baseline.

#### Secondary Outcomes

##### Fear of Negative Evaluation

The Chinese version of the 12-item Brief Fear of Negative Evaluation Scale (BFNES), developed by Leary [50] and revised by Chen [51], was used to assess individuals’ concerns about being negatively evaluated by others. It uses a 5-point Likert scale from 1 (*not at all characteristic of me*) to 5 (*extremely characteristic of me*), with higher scores indicating greater fear of negative evaluation. In this study, the BFNES had a Cronbach *α* of 0.95 at baseline.

##### Depression, Anxiety, and Stress

The Chinese version of the 21-item Depression Anxiety Stress Scales–Short Form (DASS-21), developed by Lovibond and Lovibond [52] and revised by Gong et al [53], was used to assess the severity of depressive symptoms, anxiety or autonomic arousal, and stress. It uses a 4-point Likert scale from 0 (*did not apply to me at all*) to 3 (*applied to me very much or most of the time*), with higher scores indicating greater symptom severity. In this study, the DASS-21 had a Cronbach α of 0.93 at baseline.

##### Social Anxiety Knowledge

The self-developed Social Anxiety Knowledge Test (SAKT) was used to assess participants’ knowledge of the definition, symptoms, treatment, and related aspects of social anxiety. Based on a literature review, the content was categorized into 8 domains: basic knowledge, symptoms, prevalence, age of onset, gender differences, cultural differences, risk factors, and treatment. An initial pool of 23 single-choice items (with 4 answer options each) was rated and reviewed by 7 experts for content validity, and semistructured cognitive interviews were conducted with 5 nonspecialists. After revisions based on the expert ratings and cognitive interview results, 21 items were retained. Responses were scored as correct (1 point) or incorrect (0 points), with higher total scores (maximum=21) indicating better social anxiety literacy. The SAKT has been adopted in several interventions and shown good validity to test social anxiety−related knowledge [29,54].

##### Stigmatizing Attitudes Toward Social Anxiety

The self-developed Social Anxiety Stigma Inventory (SASI) was used to assess individuals’ stigmatizing attitudes toward social anxiety. The 10-item questionnaire uses a 5-point Likert scale from 0 (*strongly disagree*) to 4 (*strongly agree*), with higher scores indicating stronger stigma. The scale demonstrated good structural validity (*χ*^2_32_^= 80.4, root mean square error of approximation=0.061, comparative fit index=0.933, Tucker-Lewis index=0.906, root mean square residual=0.048) and an internal consistency reliability of 0.75. In this study, the SASI had a Cronbach *α* of 0.86 at baseline. The SASI has been adopted in several interventions and shown good reliability and validity to assess the stigma toward social anxiety [29,54].

##### Attitudes Toward Seeking Professional Psychological Help

The Chinese version of the 10-item Attitudes Toward Seeking Professional Psychological Help Scale–Short Form (ATSPPH-SF), developed by Fischer and Farina [55] and revised by Fang et al [56], was used to assess individuals’ attitudes toward psychological help-seeking. This instrument employs a 4-point Likert scale from 0 (*strongly disagree*) to 3 (*strongly agree*), with higher scores indicating a stronger willingness to seek help. In this study, the ATSPPH-SF had an acceptable Cronbach *α* of 0.73 at baseline.

### Data Analysis

The data were analyzed following the intent-to-treat principle, including all randomized participants. The Shapiro-Wilk test was employed to assess residual normality for each scale across groups and time points. Intervention effects over time were examined using linear mixed models, which are less sensitive to missing data. Given that residuals for certain variables at specific time points were skewed and the sample size was limited, a bootstrap method with 1000 resamples was applied to estimate the linear mixed model fixed-effect parameters and their 95% CIs, thereby enhancing the robustness of statistical inferences and avoiding strict distributional assumptions [57].

Cohen *d* was calculated to determine both between-group and within-group effect sizes. Owing to baseline differences between the intervention and waitlist control groups, Morris’ baseline-adjusted formula was applied to adjust the between-group effect sizes at posttest and follow-up [58]. The effect size was based on mean pre-post change (*M_post, T_* – *M_pre, T_*) in the intervention group minus the mean pre-post change (*M_post, C_* – *M_pre, C_*), divided by the pooled pretest standard deviation (*SD_pre_*). All analyses were conducted using RStudio (version 4.5.0).


$$d=\frac{\left(M_{post,\;\;T}-M_{pre,\;\;T}\right)-\left(M_{post,\;\;C}-M_{pre,\;\;C}\right)}{SD_{pre}}$$

where the pooled standard deviation is defined as


$$SD_{pre}=\sqrt{\frac{\left(n_{T}-1\right)SD_{pre,T}^{2}+\left(n_{C}-1\right)SD_{pre,C}^{2}}{n_{T}+n_{C}-2}}$$

## Results

### Participants’ Demographic Characteristics and Baseline Scores

**Figure 1.**
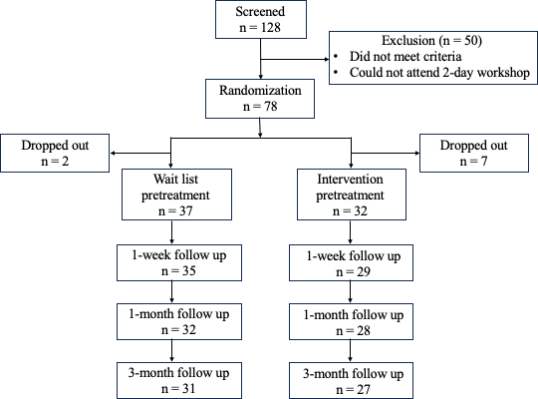
Participant flow.

The detailed flow of participants’ recruitment, allocation, and analysis is presented as Figure 1. No significant differences were found between the intervention and control groups in demographic variables or baseline outcome scores (see Table 2).

**Table 2.** Demographic characteristics and the baseline of participants.

Variable	All	Group		*t* test (*df*)	Chi-square (*df*)	*P* value
		Intervention (n=32)	Control (n=37)			
Age, mean (SD) (y)	23.16 (3.89)	22.47 (2.99)	23.76 (4.48)	−1.39 (67)	—^a^	.17
Gender, n (%)				—	0.39 (1)	.53
Man	17 (24.6)	9 (28.1)	8 (21.6)			
Woman	52 (75.4)	23 (71.9)	29 (78.4)			
Residence, n (%)				—	2.41 (2)	.30
Rural area	24 (34.8)	14 (43.8)	10 (27)			
Township	12 (17.4)	4 (12.5)	8 (21.6)			
City	33 (47.8)	14 (43.8)	19 (51.4)			
Education, n (%)				—	1.41 (2)	.495
Bachelor	40 (58)	18 (56.3)	22 (59.5)			
Master	25 (36.2)	11 (34.4)	14 (37.8)			
Doctor	4 (5.8)	3 (9.4)	1 (2.7)			
Baseline scores, mean (SD)						
SPIN^b^	—	38.97 (15.78)	37.00 (11.69)	−0.58 (67)	—	.56
BFNES^c^	—	46.19 (7.41)	45.51 (7.76)	−0.37 (67)	—	.71
DASS-21^d^	—	20.84 (10.16)	18.54 (10.53)	−0.92 (67)	—	.36
SAKT^e^	—	10.34 (2.67)	11.22 (2.18)	1.47 (67)	—	.14
SASI^f^	—	17.41 (6.71)	17.49 (6.06)	0.05 (67)	—	.96
ATSPPH-SF^g^	—	19.19 (4.64)	19.72 (4.15)	0.51 (67)	—	.61

a Not applicable.

b SPIN: Social Phobia Inventory.

c BFNES: Brief Fear of Negative Evaluation Scale.

d DASS-21: Depression Anxiety Stress Scales–Short Form.

e SAKT: Social Anxiety Knowledge Test.

f SASI: Social Anxiety Stigma Inventory.

g ATSPPH-SF: Attitudes Toward Seeking Professional Psychological Help Scale–Short Form.

### Shapiro-Wilk Normality Test

Shapiro-Wilk normality tests were conducted separately for the intervention and waitlist control groups at each time point for all outcome variables. The results showed that for both groups, the residuals of the 5 outcome variables across 4 time points were generally normally distributed (*W*=0.93‐0.98, *P*’s=.052-.902, n=35). Exceptions were observed in a few cases: the BFNES in the waitlist group at T1 (*W*=0.93, *P*<.05) and T4 (*W*=0.89, *P*=.004) and the DASS-21 in the intervention group at T2 (*W*=0.91, *P*<.05) and T4 (*W*=0.90, *P*<.05) and in the waitlist control group at T4 (*W*=0.89, *P*=.003). Overall, these findings suggest that the residual distributions did not exhibit significant skewness.

### Primary Outcomes

**Figure 2.**
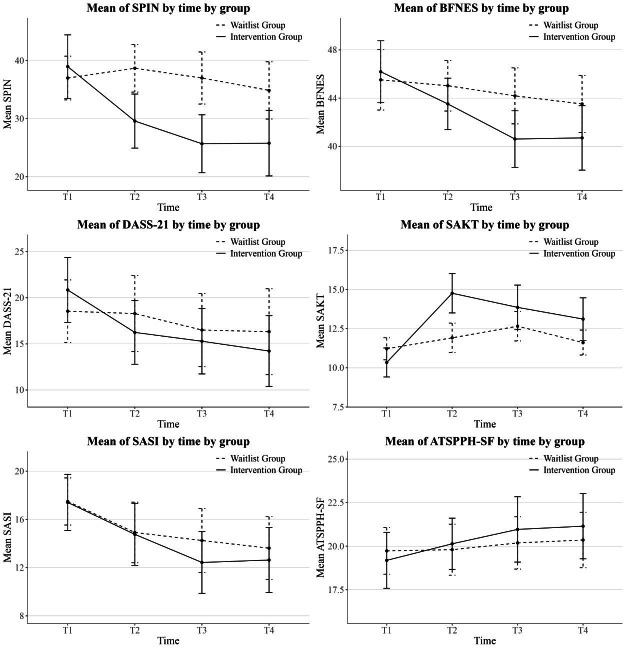
Change of outcomes. ATSPPH-SF: Attitudes Toward Seeking Professional Psychological Help Scale–Short Form; BFNES: Brief Fear of Negative Evaluation Scale; DASS-21: Depression Anxiety Stress Scales–Short Form; SAKT: Social Anxiety Knowledge Test; SASI: Social Anxiety Stigma Inventory; SPIN: Social Phobia Inventory.

According to the results (Table 3), the main effects of group and time on SPIN scores were not significant. However, the group×time interaction was significant (*β*=−4.00, 95% bootstrap CI −6.55 to −1.22), indicating that compared to the waitlist control group, the intervention group experienced a significant reduction in social anxiety over time (see Figure 2 for the trend). Moreover, the intervention group exhibited significant reductions with large effect sizes at T2 (*d*=−0.81, 95% CI −1.28 to −0.33), T3 (*d*=−0.97, 95% CI −1.44 to −0.49), and T4 (*d*=−0.81, 95% CI −1.28 to −0.33; Table 4).

**Table 3.** Results of linear mixed model analysis.

Variable	Group		Time		Group×time	
	*β*	95% boot CI	*β*	95% boot CI	*β*	95% boot CI
SPIN^a^	2.76	−4.48 to 9.19	3.26	−1.71 to 6.80	−4.00	−6.55 to −1.22
BFNES^b^	1.22	−2.50 to 4.60	0.65	−1.31 to 2.64	−1.37	−2.64 to −0.08
DASS-21^c^	2.89	−2.18 to 7.37	1.20	−1.81 to 4.21	−1.77	−3.42 to 0.17
SAKT^d^	−0.42	−1.93 to 1.10	−0.43	−1.14 to 0.38	0.62	0.05 to 1.17
SASI^e^	0.46	−2.09 to 3.43	−0.85	−2.41 to 0.62	−0.43	−1.43 to 0.62
ATSPPH-SF^f^	−0.75	−2.87 to 0.94	0.02	−1.04, 1.03	0.32	−0.39 to 1.09

a SPIN: Social Phobia Inventory.

b BFNES: Brief Fear of Negative Evaluation Scale.

c DASS-21: Depression Anxiety Stress Scales–Short Form.

d SAKT: Social Anxiety Knowledge Test.

e SASI: Social Anxiety Stigma Inventory.

f ATSPPH-SF: Attitudes Toward Seeking Professional Psychological Help Scale–Short Form.

**Table 4.** Means, Standard deviations, and Cohen *d* for outcome variables by group and time^a^.

Variable	Intervention group		Control group		*d*_b-g_^c^ (95% CI)
	Mean (SD)	*d*_w-g_^b^ (95% CI)	Mean (SD)	*d*_w-g_	
SPIN^d^					
T1	38.97 (15.78)	—^e^	37.00 (11.69)	—	0.14 (–0.33 to 0.62)
T2	29.31 (13.16)	0.71 (–1.07 to –0.31)	37.59 (12.98)	0.10 (–0.27 to 0.41)	–0.81 (–1.28 to –0.33)
T3	26.03 (13.44)	–0.91 (–1.27 to –0.51)	36.46 (12.71)	–0.08 (–0.45 to 0.29)	–0.97 (–1.44 to –0.49)
T4	25.78 (14.96)	–0.89 (–1.24 to –0.53)	34.87 (14.00)	–0.11 (–0.48 to 0.25)	–0.81 (–1.28 to –0.33)
BFNES^f^					
T1	46.19 (7.41)	—	45.51 (7.76)	—	0.009 (–0.39 to 0.56)
T2	42.81 (6.19)	–0.52 (–0.81 to –0.17)	44.57 (6.48)	–0.01 (–0.44 to 0.32)	–0.29 (–0.76 to 0.19)
T3	40.16 (6.61)	–0.74 (–1.09 to –0.40)	43.00 (7.00)	–0.12 (–0.53 to 0.28)	–0.56 (–1.03 to –0.09)
T4	40.7 (7.08)	–0.76 (–1.04 to –0.42)	43.52 (6.72)	–0.20 (–0.52 to 0.24)	–0.46 (–0.93 to 0.01)
DASS-21^g^					
T1	20.84 (10.16)	—	18.54 (10.53)	—	0.22 (–0.25 to 0.70)
T2	16.66 (9.24)	–0.63 (–1.01 to –0.26)	17.97 (12.17)	0.02 (–0.35 to 0.32)	–0.42 (–0.89 to 0.05)
T3	15.41 (9.85)	–0.71 (–1.17 to –0.31)	16.14 (10.82)	–0.22 (–0.55 to 0.15)	–0.34 (–0.81 to 0.13)
T4	14.22 (10.18)	–0.78 (–1.19 to –0.36)	16.32 (13.23)	–0.07 (–0.41 to 0.31)	–0.43 (–0.90 to 0.05)
SAKT^h^					
T1	10.34 (2.67)	—	11.22 (2.19)	—	–0.36 (–0.84 to 0.12)
T2	14.19 (3.82)	1.21 (0.67 to 1.70)	11.65 (2.98)	0.23 (–0.12 to 0.50)	1.53 (1.06 to 2.01)
T3	13.31 (3.92)	0.98 (0.48 to 1.48)	12.46 (2.86)	0.40 (0.01 to 0.76)	0.86 (0.38 to 1.33)
T4	13.11 (3.59)	0.77 (0.37 to 1.18)	11.61 (2.26)	0.11 (–0.30 to 0.47)	0.98 (0.51 to 1.45)
SASI^i^					
T1	17.41 (6.71)	—	17.49 (6.06)	—	0.01 (–0.49 to 0.46)
T2	14.84 (6.73)	–0.44 (–0.85 to –0.05)	14.51 (7.76)	–0.55 (–0.86 to –0.16)	–0.01 (–0.49 to 0.46)
T3	12.88 (7.09)	–0.78 (–1.19 to –0.38)	14.27 (8.15)	–0.50 (–0.85 to –0.06)	0.27 (–0.75 to 0.20)
T4	12.63 (7.14)	–0.68 (–0.96 to –0.23)	13.61 (7.41)	–0.74 (–1.15 to –0.19)	0.14 (–0.62 to 0.33)
ATSPPH-SF^j^					
T1	19.19 (4.64)	—	19.73 (4.15)	—	0.12 (–0.60 to 0.35)
T2	19.84 (3.96)	0.19 (–0.19 to 0.56)	20.03 (4.41)	0.15 (–0.25 0.42)	0.20 (–0.27 to 0.67)
T3	20.69 (4.86)	0.34 (–0.10 to 0.68)	20.32 (4.36)	0.14 (–0.20, 0.51)	0.30 (–0.17 to 0.77)
T4	21.15 (4.97)	0.39 (0.01 to 0.75)	20.35 (4.52)	0.31 (–0.09, 0.70)	0.31 (–0.17 to 0.78)

a Negative values reflect reductions from the baseline. Bootstrapped 95% CIs are reported in parentheses.

b Within-group Cohen *d.*

c Between-group Cohen *d.*

d SPIN: Social Phobia Inventory.

e Not available.

f BFNES: Brief Fear of Negative Evaluation Scale.

g DASS-21: Depression Anxiety Stress Scales–Short Form.

h SAKT: Social Anxiety Knowledge Test.

i SASI: Social Anxiety Stigma Inventory.

j ATSPPH-SF: Attitudes Toward Seeking Professional Psychological Help Scale–Short Form.

### Secondary Outcomes

The main effects of group and time on BFNES scores were not significant. However, the group×time interaction effect was significant (*β*=−1.37, 95% bootstrap CI −2.64 to −0.08), indicating that compared to the waitlist control group, the intervention group experienced a significant reduction in fear of negative evaluation over time. However, this reduction was statistically significant only at T3, with a moderate effect size (*d*=−0.56, 95% CI −1.03 to −0.09).

For DASS-21 scores, neither the main effects of group and time nor the group×time interaction effect was significant (*β*=-1.77, 95% bootstrap CI −3.42 to 0.17). This indicates that no significant differences existed between the groups in terms of depression, anxiety, or stress symptom trajectories over time.

The main effects of group and time on SAKT scores were not significant. However, the group×time interaction effect was significant (*β*=.62, 95% bootstrap CI 0.05 to 1.17), indicating that compared to the waitlist control group, the intervention group showed a significant increase in social anxiety knowledge over time. Large effect sizes were observed at T2 (*d*=1.53, 95% CI 1.06-2.00), T3 (*d*=0.86, 95% CI 0.38-1.33), and T4 (*d*=0.98, 95% CI 0.51-1.45).

For SASI scores, the main effects of group and time, as well as the group×time interaction (*β*=−.43, 95% bootstrap CI −1.43 to 0.62), were not significant. This suggests that there were no significant differences between the groups in terms of social anxiety stigma over time. Similarly, no significant effects were found for ATSPPH-SF scores. The pattern of results mirrored that of the SASI, indicating no significant between-group differences in attitudes toward seeking professional psychological help over time.

## Discussion

### General Findings

This study examined the effectiveness of a CBT-based MBPG delivered via videoconference for addressing social anxiety in university students. The results showed that the intervention led to significant improvements in social anxiety, including reductions in participants’ fear of negative evaluation over a certain period and a marked increase in social anxiety knowledge. However, no significant improvements were observed in emotional distress (ie, depression, anxiety, and stress as measured by the DASS-21), social anxiety stigma, or attitudes toward seeking professional psychological help.

First, compared to the waitlist control group, participants in the intervention group reported significant reductions in social anxiety, with large effect sizes observed in both the short term (1-week posttest and 1-month follow-up) and longer term (3-month follow-up). Moreover, fear of negative evaluation significantly decreased 1 month after the intervention, consistent with the study’s hypotheses. A recent meta-analysis reported that various forms of remote CBT yield medium-to-large effect sizes in treating social anxiety [19]. While this study did not directly compare videoconference delivery to other remote CBT modalities, the observed effect sizes align with those reported in prior research on remote CBT for social anxiety, with relatively durable effects. This further suggests that core CBT techniques, particularly cognitive restructuring and behavioral experiments targeting automatic thoughts, can be effectively delivered in both in-person and remote formats, likely without significant differences in efficacy.

Second, this study employed a massed brief intervention, delivering 12 hours of intervention intensively over 2 consecutive days. High-frequency delivery of this kind has been shown to sustain participants’ motivation and engagement [36]. The selected intervention components in this study are well-established techniques for reducing social anxiety, and the massed format enables tighter integration across modules, allowing therapeutic gains to accumulate within a short period [59]. Therefore, despite involving fewer sessions than full-protocol interventions, this brief intervention yielded robust therapeutic outcomes. Given that fear of negative evaluation is a core cognitive mechanism in the development and maintenance of social anxiety [60], its reduction further supports the intervention’s efficacy. However, in this study, improvement in fear of negative evaluation was significant only at the 1-month follow-up. This suggests that the observed reduction in social anxiety may involve additional mediating mechanisms beyond cognitive change alone, which warrants further investigation in future research.

However, the intervention did not have significant effects on anxiety, depression, or stress, which was inconsistent with the study’s hypotheses. Cognitive restructuring, a core component of this intervention, is a widely applicable CBT technique and is theoretically effective for treating anxious or depressive symptoms as well [61]. Moreover, prior studies using videoconferencing group CBT for social anxiety have reported significant reductions in DASS-21 scores [40,41]. A 1-day CBT-based workshop for secondary vocational students with social anxiety also significantly reduced DASS-21 scores, with moderate effect sizes [29]. However, not all prior findings are consistent: an in-person group CBT intervention for social anxiety using a full treatment protocol found no improvements in depressive symptoms [62]. Taken together, the absence of significant change in DASS-21 scores in this study may suggest that the MBPG via videoconference has limitations in the intervention’s depth. Compared to full-protocol CBT, the brief, massed format involves fewer techniques and a considerably shortened duration. Specifically, spaced CBT interventions commonly provide opportunities for participants to practice newly learned skills between weekly sessions through homework assignments, thereby consolidating gains and facilitating symptom improvement [63]. Although the MBPG also allowed participants to practice during sessions and lunch breaks, these chances were still limited, which in turn constrained the extent to which negative beliefs and cognitive biases could be corrected. This may inherently limit its capacity to address broader emotional symptom domains beyond social anxiety or reduce the extent of therapeutic change achievable within participants. In addition, the DASS-21 primarily assesses physiological arousal and subjective emotional experiences, rather than cognitive content. As this intervention targeted participants’ cognitive biases, improvements in social anxiety were likely driven by cognition-focused changes. Such changes, however, are not readily captured by the DASS-21. Although the DASS-21 is commonly used in social anxiety interventions, it may be less suitable for detecting the specific types of cognitive changes targeted in this study.

The outcomes related to mental health literacy on social anxiety were somewhat complex, with the corresponding hypotheses only partially supported. Participants’ knowledge of social anxiety significantly improved, reflecting the most direct impact of the psychoeducational component and aligning with the study’s hypotheses. However, no significant changes were observed in social anxiety stigma or in attitudes toward seeking professional help. Prior research has shown that when individuals’ mental health literacy regarding social anxiety improves, they tend to adopt a more objective view of the condition, experience reduced stigma, and show a greater willingness to seek professional help [64]. Furthermore, Cui et al [54] conducted a 6-week, in-person psychoeducation group with the same measurements and found significant improvements in knowledge, stigma, and help-seeking attitudes. We believe several factors may account for the discrepancy between their findings and ours. First, disclosure among people with mental illness is an effective strategy to manage stigma [65], yet opportunities for interpersonal contact were highly constrained in the online format compared with in-person groups. This limitation likely hindered meaningful changes in stigma. Second, help-seeking attitudes are influenced by multiple factors, including mental health literacy and perceived service accessibility [66], and improving these attitudes requires shifts in participants’ deeper beliefs and motivations [64]. However, participants in the online workshop were recruited from diverse regions across the country, resulting in variability in access to mental health resources. For participants from low-resource backgrounds, severely limited perceived accessibility of services may have constrained improvements in help-seeking attitudes. Therefore, no significant change in help-seeking attitudes was observed at the group level. Nonetheless, these findings tentatively suggest that improvements in social anxiety symptoms may occur independently of changes in stigmatizing attitudes toward social anxiety.

### Implications, Limitations, and Future Research

This study has several notable strengths. First, it employs a videoconference platform to deliver a new format of intervention, that is, the MBPG, which offers numerous advantages. The videoconferencing delivery format overcomes geographical barriers, increasing access to evidence-based interventions. At the same time, however, online delivery inevitably reduces opportunities for in-person exposure. It must be carefully considered when selecting intervention techniques, as the effectiveness of exposure-based strategies may be more substantially limited. In this intervention, both the cognitive restructuring and behavioral experiments were cognition-targeted techniques that do not rely on exposure in vivo, thereby minimizing the potential impact of the videoconferencing format on treatment effects. The massed format—delivered over a short, tightly scheduled period—may improve efficiency and reduce dropout. Moreover, the psychoeducational group format accommodates more participants per session, and its highly structured, slide-based delivery reduces the demands on group leaders while ensuring treatment fidelity. Finally, the use of a randomized controlled trial design provides preliminary yet promising evidence for the efficacy of this intervention model.

The study also has some limitations. First, the sample size was relatively small, and we conducted our intervention only in the nonclinical sample. Future research should expand upon these findings by conducting large-scale, multicenter randomized controlled trials in both clinical and community samples to further evaluate the effects of the MBPG-based program for social anxiety. Second, videoconferencing-based CBT has inherent constraints compared to the face-to-face format. These include increased exposure to distractions, reduced attentional engagement, and limited ability for leaders to observe and monitor participant cues [67], which further reduce opportunities for direct contact and spontaneous communication both between the leaders and participants and among participants themselves. Such limitations may reduce intervention efficacy, warranting improved technological solutions and content organization to address these challenges. In addition, compared to traditional treatments, the massed brief intervention format offers limited opportunities for participants to practice newly acquired cognitive skills. As a result, these skills may not be sufficiently reinforced, which in turn constrains the depth of therapeutic change. Future iterations of the intervention protocol should incorporate additional strategies to address this limitation. Finally, the follow-up period was limited to 3 months. Longer-term follow-up (eg, 6 months, 1 year, or beyond) is necessary to assess the maintenance of treatment gains over time.

We finally derived several implications from this intervention for future research. First, although online delivery is convenient and lowers access barriers, it is important to select a platform that supports essential interactive features, particularly breakout rooms and spaces that allow participants to communicate more freely, which may partially mitigate the limitations associated with reduced in-person exposure. Second, because the content of this workshop was delivered in a highly structured, slide-based format, future dissemination could broaden not only the reach of the program but also the range of potential leaders. Beyond mental health professionals, trained paraprofessionals or nonspecialists (eg, peer counselors or students majoring in psychology or related fields) could deliver the intervention using standardized materials, such as guidance menus and slide decks, thereby enhancing the accessibility of mental health care.

### Conclusion

This study demonstrates that a videoconferencing CBT-based massed brief psychoeducational group can produce meaningful and sustained improvements in social anxiety among university students. Future research should focus on scaling up this model through larger randomized controlled trials, adapting it for more diverse populations, and exploring the feasibility of delivery by nonspecialist leaders.

## Supplementary material

10.2196/79825 Checklist 1 CONSORT-eHEALTH checklist (V 1.6.1).

## Abbreviations

ATSPPH-SF: Attitudes Toward Seeking Professional Psychological Help Scale−Short Form
BFNES: Brief Fear of Negative Evaluation Scale
CBT: cognitive-behavioral therapy
DASS-21: Depression Anxiety Stress Scales
MBPG: Massed Brief Psychoeducational Group
SAKT: Social Anxiety Knowledge Test
SASI: Social Anxiety Stigma Inventory
SPIN: Social Phobia Inventory
